# Reversible splenial lesion syndrome in children: a retrospective study of 130 cases

**DOI:** 10.3389/fneur.2023.1241549

**Published:** 2023-09-05

**Authors:** Hui Chen, Xiongying Yu, Yong Chen, Huaping Wu, Zhuqiang Wu, Jianmin Zhong, Zhenyu Tang

**Affiliations:** ^1^Nanchang University, Nanchang, China; ^2^Department of Neurology, Children's Hospital of Jiangxi Province, Nanchang, China; ^3^Department of Magnetic Resonance, Children's Hospital of Jiangxi Province, Nanchang, China; ^4^Department of Neurology, The Second Affiliated Hospital of Nanchang University, Nanchang University, Nanchang, China

**Keywords:** reversible splenial lesion syndrome, children, magnetic resonance imaging, infection, convulsions

## Abstract

**Background:**

Reversible splenial lesion syndrome (RESLES) is a new clinico-radiological syndrome. We retrospectively analyzed the clinical features of 130 children with RESLES in China, which is the largest case series available in the literature.

**Methods:**

The clinical data of children diagnosed as RESLES in Jiangxi Provincial Children's Hospital between 2017 and 2023 were retrospectively analyzed. The 130 cases were divided into two groups: ≤ 3 years old group (group A) (*n* = 83) and > 3 years old group (group B) (*n* = 47). The chi-squared test or Fisher's test was used to evaluate the data.

**Results:**

The vast majority of patients (127/130 cases, 97.7%) had prodromal symptoms of infection. Preceding infections of the gastrointestinal tract were statistically more significant in group A (60/83, 72.3%) than in group B (11/47, 23.4%) (*P* < 0.05). Preceding infections of the respiratory tract were statistically more significant in group B (33/47, 70.2%) than in group A (17/83, 20.5%) (*P* < 0.05). Seizures were statistically more significant in group A (82/83, 98.8%) than in group B (24/47,51.1%) (*P* < 0.05). The disturbance of consciousness and headache/dizziness were statistically more significant in group B (27/47, 57.4%; 37/47, 78.7%) than in group A (3/83, 3.6%; 1/83, 1.2%), respectively (*P* < 0.05). Convulsions with mild gastroenteritis (CwG) were statistically more significant in group A (50/83, 60.2%) than in group B (8/47, 17.0%) (*P* < 0.05). However, encephalitis/encephalopathy was statistically more significant in group B (20/47, 42.6%) than in group A (10/83, 12.0%) (*P* < 0.05). MRI showed cytotoxic edema in typical locations (RESLES type-1 limited to the splenium of the corpus callosum and RESLES type-2 spread to the entire corpus callosum, adjacent white matter, or both). There was full recovery of the lesions of MRI in all cases from 3 days to 50 days after the initial examinations. All the children showed normal neurodevelopment.

**Conclusion:**

Infection was the most common cause of RESLES. Infections of the gastrointestinal tract are common in ≤ 3 years old children, while infections of the respiratory tract are common in >3 years old children. Younger patients are more likely to develop convulsions, and older children were more likely to have symptoms with disturbance of consciousness and headache/dizziness. RESLES has characteristic MRI manifestations and a good prognosis.

## 1. Introduction

Mild encephalitis/encephalopathy with a reversible splenial lesion (MERS) is a clinical and radiological syndrome that was originally reported by Tada et al. in 2004 (1). The syndrome is characterized by a reversible lesion with homogenously reduced diffusion in the corpus callosum (at least involving the splenium), sometimes associated with symmetrical white matter lesions, which is identified by magnetic resonance imaging (MRI) (2). Because the use of MRI has become widespread, various cases accompanied by MERS have been reported. In 2011, Garcia-Monco et al. described the syndrome in detail and proposed a new term called “reversible splenial lesion syndrome (RESLES)” (3). RESLES can be divided into two types according to the imaging findings of how the lesion spreads. RESLES type-1 is the typical type and shows an isolated splenium of a corpus callosum (SCC) lesion. On the contrary, RESLES type-2 is rare, and it shows extensive white matter and/or entire callosal lesions (4–7).

RESLES typically presents with mild central nervous system disturbances following prodromal symptoms, such as fever, cough, vomiting, and/or diarrhea. Neurological symptoms of RESLES are characterized by seizure, confusion, delirium, abnormal speech, muscle weakness, ophthalmoplegia, facial nerve paralysis, blurred vision, and headache. Affected patients usually recover completely without any sequelae within a month after the onset of neurological symptoms (7–9). Although RESLES has been reported in patients with a variety of conditions, its definitive pathogenesis remains unclear. Potential causes include infection, seizures, antiepileptic drug use or withdrawal, metabolic disturbances, demyelinating neuropathies, vitamin B12 deficiency, malnutrition, high-altitude cerebral edema, systemic lupus erythematosus, Kawasaki disease, and malignancy (3, 10–20). In recent years, although there were more and more reports on RESLES, most of them were case reports, literature review, and small sample reports. Here, we retrospectively analyzed the features of 130 children with RESLES, which is the largest case series available in the literature. Meanwhile, these cases were followed up, and our study compared the clinical characteristics of different age groups, and no similar studies have been conducted in the past, which has guiding significance for clinical diagnosis, treatment, and management of RESLES.

## 2. Patients and methods

### 2.1. Patients

In the retrospective study, clinical data of children diagnosed with RESLES in the Department of Neurology of Jiangxi Provincial Children's Hospital from September 2017 to April 2023 were collected. The inclusion criteria for the study were as follows (3, 8): (1) children presented with neurological symptoms; (2) MRI showed cytotoxic edema in typical locations (RESLES type-1 limited to the SCC and RESLES type-2 spread to the entire corpus callosum, adjacent white matter, or both); and (3) both the clinical symptoms and imaging findings were reversible. The exclusion criteria for our study were as follows: (1) follow-up was not available; (2) patients with acute disseminated encephalopathy and other common demyelinating disorders involving the corpus callosum, such as acute disseminated encephalomyelitis (ADEM) and neuromyelitis optica spectrum disorders (NMOSD); and (3) patients who were not reviewed for cranial MRI during follow-up. This study was approved by the ethics committee of Jiangxi Provincial Children's Hospital in China. Written informed consent was obtained from the children's guardian.

### 2.2. Methods

Clinical data including age, gender, prodromal symptoms, central nervous system symptoms, results of laboratory examination, electroencephalogram (EEG) findings, MRI findings, treatment, and patient outcomes were collected. The results of laboratory examination included electrolytes, liver function, myocardial enzymes, cerebrospinal fluid (CSF), and pathogens. Analyses of EEG and MRI were performed by qualified electroencephalogists and radiologists, respectively. Follow-up was conducted through telephone or return visit for a period of 2–69 months. The neurodevelopment was assessed according to medical history and clinical judgment.

Statistical analysis was performed through IBM SPSS Statistics 21. The enumeration data were expressed as numbers and percentages *n* (%), and the chi-square test or Fisher's test was used to evaluate the enumeration data. A *P*-value of <0.05 was regarded as statistically significant.

## 3. Results

### 3.1. General information

A total of 137 patients had central nervous system symptoms, and MRI showed cytotoxic edema in the corpus callosum. However, of these, two patients were eventually diagnosed with ADEM, while five patients were not followed up. Therefore, a total of 130 patients were enrolled in the study. Of the 130 patients, 77 were male patients and 53 were female patients. The onset age ranged from 8 months to 153 months, with a median age of 32 months (Table 1). Because of different clinical manifestations in different onset age years, 130 cases were divided into two groups: ≤ 3 years old group (*n* = 83) and >3 years old group (*n* = 47). The ≤ 3 years old group and >3 years old group were called group A and group B, respectively. There were 51 male patients (61.4%) and 32 female patients (38.6%) in group A and 26 male patients (55.3%) and 21 female patients (44.7%) in group B, respectively. There was no significant difference in gender between the two groups (*P* > 0.05) (**Table 3**).

**Table 1:** General and clinical characteristics of patients.

**Variable**	**Data *n* (%)**
**Onset age (median age)**	8–153 months (32 months)
**Gender**	
Male	77 (59.2)
Female	53 (40.8)
**Prodromal symptoms**	
Respiratory tract infection	50 (38.5)
Digestive tract infection	71 (54.6)
Both respiratory and digestive tract infection	6 (4.6)
Without prodromal symptoms	3(2.3)
**Neurological symptoms**	
Disturbance of consciousness	30 (23.1)
Seizure	106 (81.5)
Headache or dizziness	38 (29.2)
Limb weakness	3 (2.3)
Dysarthria	2 (1.5)
Ataxia	5 (3.8)
Abnormal behavior	3 (2.3)
**Overall neurological syndrome**	
CwG	58 (44.6)
FS (CFS)^ý^	16 (12.3)
Epilepsy	12 (9.2)
Encephalitis/Encephalopathy	30 (23.1)
Respiratory or digestive tract infection	14 (10.8)
**Treatment**	
Mannitol	43 (33.1)
Dexamethasone	9 (6.9)
**Follow-up**	
Duration (median)	2–69 months (39 months)
Normal neurodevelopment	130 (100)
Lesion disappearance (MRI) (median)	3–50 days (8days)
Sequelae	0 (0)

CwG, convulsions with mild gastroenteritis; FS, febrile seizure; CFS, complex febrile seizure; MRI, magnetic resonance imaging.

^ý^Because MRI is not routinely recommended for simple febrile seizure, all FS patients in this study were complex febrile seizure.

### 3.2. Clinical manifestation

The vast majority of patients (127/130 cases, 97.7%) had prodromal symptoms of infection. More than half of the patients (71/130 cases, 54.6%) had preceding infections of the gastrointestinal tract, such as fever, vomiting, abdominal pain, and diarrhea predominantly. Fifty cases (38.5%) had preceding infections of the respiratory tract, such as fever, cough, runny nose, stuffy nose, and sore throat predominantly. Of all the cases, six cases (4.6%) had both preceding infections of the gastrointestinal tract and the respiratory tract. However, three cases (2.3%) did not have any prodromal symptoms (Table 1). Of the 130 patients, 107 (82.3%) had fever within 1 week before the onset of neurological symptoms. In group A, more than half of the patients (60/83 cases, 72.3%) had preceding infections of the gastrointestinal tract. However, only 11 cases (23.4%) had preceding infections of the gastrointestinal tract in group B. The comparison between the two groups was statistically significant (*P* < 0.05). In group B, more than half of the patients (33/47 cases, 70.2%) had preceding infections of the respiratory tract. However, only 17 cases (20.5%) had preceding infections of the respiratory tract in group A. The comparison between the two groups was statistically significant (*P* < 0.05) (**Table 3**). The vast majority of patients (106/130 cases, 81.5%) developed seizures. Partial patients were accompanied by other neurological symptoms, including headache or dizziness (38 cases), disturbance of consciousness (30 cases), ataxia (5 cases), abnormal behavior (3 cases), limb weakness (3 cases), and dysarthria (2 cases) (Table 1). In group A, almost all the patients (82/83 cases, 98.8%) developed seizures. However, in group B, only 24 cases (51.1%) developed seizures. The comparison between the two groups was statistically significant (*P* < 0.05) (**Table 3**). In group B, disturbance of consciousness occurred in 27 cases (57.4%), and more than half of the patients (37/47 cases, 78.7%) had headache or dizziness. However, in group A, only three cases (3.6%) had disturbance of consciousness, and only one case (1.2%) had headache or dizziness. The comparison between the two groups was statistically significant (*P* < 0.05) (**Table 3**). The clinical syndrome that was associated with the 130 RESLES was as follows (Table 1): 58 (44.6%) convulsions with mild gastroenteritis (CwG), 30 (23.1%) encephalitis/encephalopathy, 16 (12.3%) febrile seizure (FS), 12 (9.2%) epilepsy, and 14 (10.8%) respiratory or digestive tract infection. In group A, more than half of the patients (50/83 cases, 60.2%) and 10 cases (12.0%) suffered from CwG and encephalitis/encephalopathy, respectively. However, in group B, 8 cases (17.0%) and 20 cases (42.6%) suffered from CwG and encephalitis/encephalopathy, respectively. The comparison between the two groups was statistically significant (*P* < 0.05) (**Table 3**).

### 3.3. Laboratory examinations

Of all the cases, 82 patients had clinically proven pathogen infection, including rotavirus in 44 cases (33.8%), mycoplasma pneumoniae in 14 cases (10.8%), influenza virus in 9 cases (6.9%), respiratory syncytial virus in 9 cases (6.9%), Epstein–Barr virus in 4 cases (3.1%), enterovirus in 1 case (0.8%), and severe acute respiratory syndrome coronavirus 2 (SARS-CoV-2) in 1 case (0.8%), respectively (Table 2). In group A, 39 cases (47.0%) had infection of rotavirus and 5 cases (10.6%) in group B. The comparison between the two groups was statistically significant (*P* < 0.05) (Table 3). In group B, 10 cases (21.3%) had infection of mycoplasma pneumoniae and 4 cases (4.8%) in group A. The comparison between the two groups was statistically significant (*P* < 0.05) (Table 3).

**Table 2:** Summary of auxiliary examination of all cases.

**Variable**	**Data *n* (%)**
**Pathogen infection**	
Rotavirus	44 (33.8)
Mycoplasma pneumoniae	14 (10.8)
Respiratory syncytial virus	9 (6.9)
Influenza virus	9 (6.9)
Epstein–Barr virus	4 (3.1)
Enterovirus	1 (0.8)
SARS-CoV-2	1 (0.8)
Unknown	48 (36.9)
Abnormal liver function	14 (10.8)
Abnormal myocardial enzyme	16 (12.3)
Hyponatremia (<135 mmol/L)	30 (23.1)
Abnormal CSF (108 patients who received lumbar puncture examinations)	6 (5.6)
Abnormal EEG	79 (60.8)
**Abnormal MRI**	
Type I	125 (96.2)
Type II	5 (3.8)

SARS-CoV-2, severe acute respiratory syndrome coronavirus 2; CSF, cerebrospinal fluid; EEG, electroencephalogram; MRI, magnetic resonance imaging.

**Table 3:** Comparison of general and clinical characteristics in patients with different ages (≤ 3 years and > 3 years old group).

**Variable *n* (%)**	**≤ 3 years old (*n* = 83) Group A**	**>3 years old (*n* = 47)** **Group B**	**P-value**
**Gender**			
Male	51 (61.4)	26 (55.3)	0.495
Female	32 (38.6)	21 (44.7)	
**Prodromal symptoms**			
Respiratory tract infection	17 (20.5)	33 (70.2)	0.000
Digestive tract infection	60 (72.3)	11 (23.4)	0.000
Both respiratory and digestive tract infection	4 (4.8)	2 (4.3)	1.000^ý^
Without prodromal symptoms	2 (2.4)	1 (2.1)	1.000^ý^
**Neurological symptoms**			
Disturbance of consciousness	3 (3.6)	27 (57.4)	0.000
Seizure	82 (98.8)	24 (51.1)	0.000
Headache or dizziness	1 (1.2)	37 (78.7)	0.000
Limb weakness	2 (2.4)	1 (2.1)	1.000^ý^
Dysarthria	1 (1.2)	1 (2.1)	1.000^ý^
Ataxia	4 (4.8)	1 (2.1)	0.653^ý^
Abnormal behavior	1 (1.2)	2 (4.3)	0.296^ý^
**Overall neurological syndrome**			
CwG	50 (60.2)	8 (17.0)	0.000
FS (CFS)	10 (12.0)	6 (12.8)	0.905
Epilepsy	9 (10.8)	3 (6.4)	0.535^ý^
Encephalitis/ encephalopathy	10 (12.0)	20 (42.6)	0.000
Respiratory or digestive tract infection	4 (4.8)	10 (21.3)	0.004
**Pathogen infection**			
Rotavirus	39 (47.0)	5 (10.6)	0.000
Mycoplasma pneumoniae	4 (4.8)	10 (21.3)	0.004
Respiratory syncytial virus	8 (9.6)	1 (2.1)	0.155^ý^
Influenza virus	6 (7.2)	3 (6.4)	1.000^ý^
Epstein–Barr virus	3 (3.6)	1 (2.1)	1.000^ý^
Enterovirus	1 (1.2)	0 (0.0)	1.000^ý^
SARS-CoV-2	1 (1.2)	0 (0.0)	1.000^ý^
Unknown	21 (25.3)	27 (57.4)	0.000
Abnormal liver function	7 (8.4)	7 (14.9)	0.254
Abnormal myocardial enzyme	11 (13.3)	5 (10.6)	0.663
Hyponatremia (<135 mmol/L)	18 (21.7)	12 (25.5)	0.617
Abnormal EEG	49 (59.0)	30 (63.8)	0.591
**Abnormal MRI**			
Type I	80 (96.4)	45 (95.7)	1.000^ý^
Type II	3 (3.6)	2 (4.3)	

CNS, central nervous system; CwG, convulsions with mild gastroenteritis; FS, febrile seizure; CFS, complex febrile seizure; SARS-CoV-2, severe acute respiratory syndrome coronavirus 2; MRI, magnetic resonance imaging; CSF, cerebrospinal fluid; EEG, electroencephalogram.

^ý^Fisher's test.

Elevated hepatic enzyme (alanine aminotransferase 40–155 U/L and aspartate aminotransferase 60–148 U/L) was found in 14 cases (10.8%), and elevated myocardial enzyme (creatine kinase 281–1,412 U/L) was found in 16 cases (12.3%). There was no significant difference between the two groups (*P* > 0.05) (Tables 2, 3).

Of all the cases, hyponatremia was presented in 30 patients (128–134 mmol/L), including 18 cases (21.7%) in group A and 12 cases (25.5%) in group B. There was no significant difference between the two groups (*P* > 0.05) (Tables 2, 3).

Of the 108 patients who received lumbar puncture examinations, only six cases (5.6%) showed abnormal results, which were characterized by increased CSF cell count (six cases, white blood cells: 35–156 × 10^6^/L, the cell classification showed that monocytes were predominant) and the increased CSF protein (one case, 800 mg/L). The sugar and chloride of CSF were normal (Table 2).

### 3.4. EEG findings

EEG examination of 79 cases (60.8%) showed abnormal EEG (Table 2), which was characterized by epileptiform discharges (7 cases), and the slow-down EEG background (72 cases). There was no significant difference in EEG between the two groups (*P* > 0.05) (Table 3).

### 3.5. MRI findings

**Figure 1:**
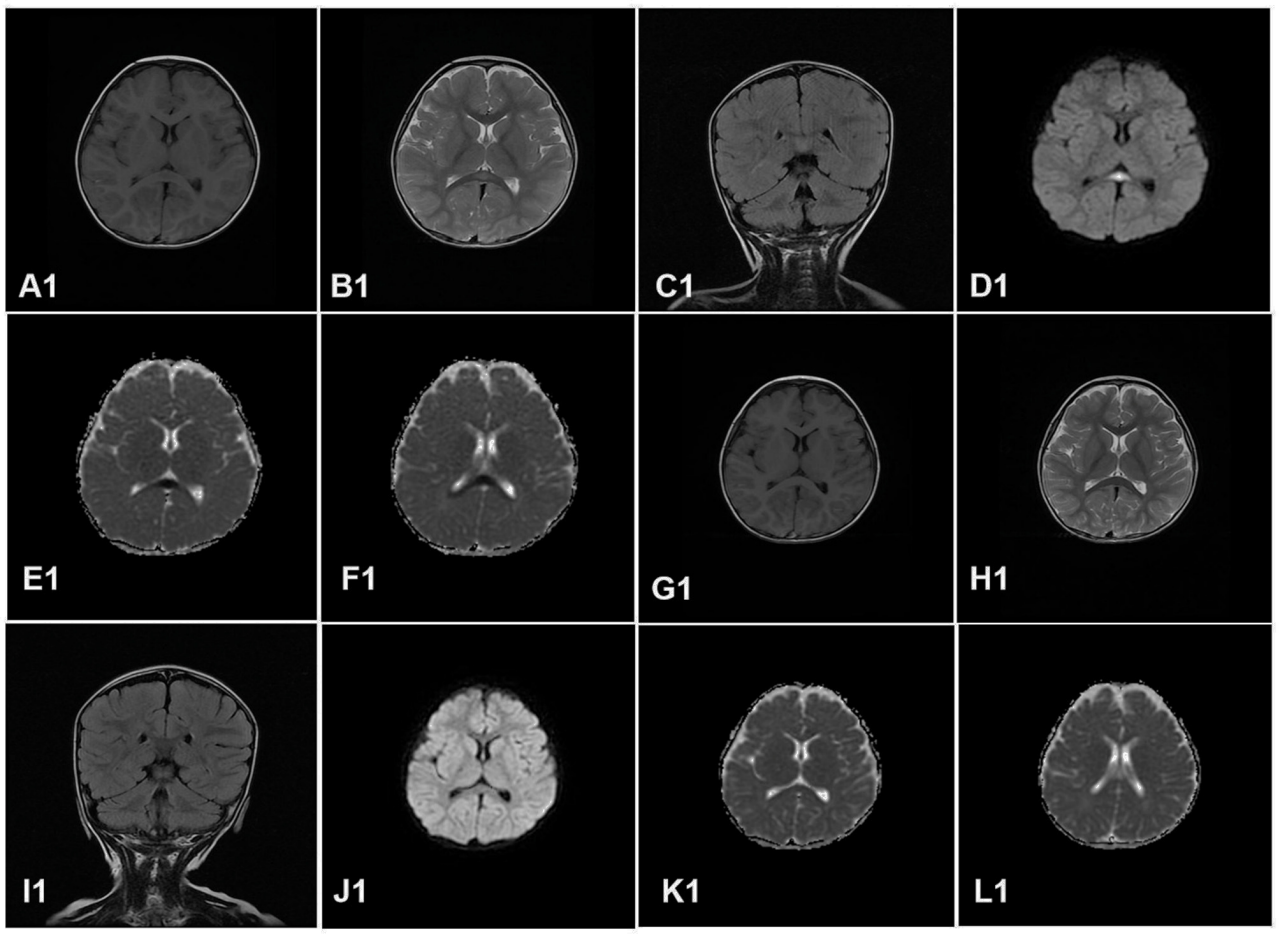
A case of RESLES type-1. Cranial MRI revealed an isolated lesion in the splenium of the corpus callosum on day 4. **(A1–F1)**: slightly hypointense on T1WIs **(A1)**; hyperintense signal on T2WIs **(B1)**, FLAIR images **(C1)**, and DWIs **(D1)**; low value on apparent diffusion coefficient (ADC) maps **(E1, F1)**. Cranial MRI revealed that all the lesions had disappeared completely on day 9 after treatment **(G1–L1)**.

**Figure 2:**
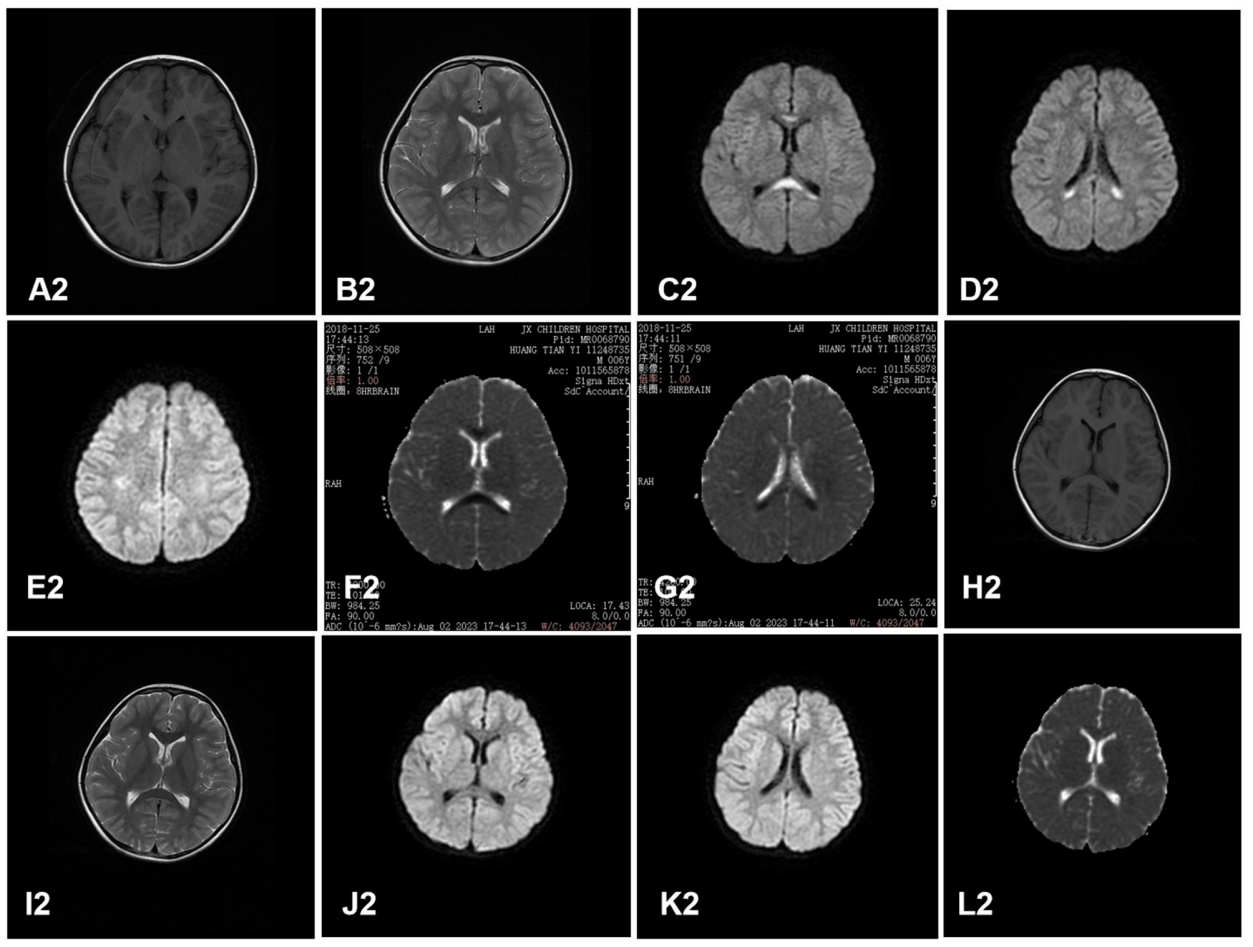
A case of RESLES type-2. Cranial MRI revealed a lesion in the entire corpus callosum, adjacent peri ventricular, and bilateral semi-ovoid center on day 2. **(A2–G2)**: slightly hypointense on T1WIs **(A2)**, hyperintense signal on T2WIs **(B2)** and DWIs **(C2, D2, E2)**, and low value on ADC maps **(F2, G2)**. Cranial MRI revealed that all the lesions had disappeared completely on day 11 after treatment **(H2–L2)**.

All the cases received the first cranial MRI examination at 1–7 days after the onset of neurological symptoms. MRI showed cytotoxic edema in typical locations (RESLES type-1 limited to the SCC and RESLES type-2 spread to the entire corpus callosum, adjacent white matter, or both). Of the 130 patients, 125 cases (96.2%) were RESLES type-1 and only 5 cases (3.8%) were RESLES type-2. These lesions were characterized by hyperintense signal on T2-weighted images (T2WIs), fluid-attenuated inversion recovery (FLAIR) images, and diffusion-weighted images (DWIs) and slightly hypointense on T1-weighted images (T1WIs) (Table 2, Figures 1, 2).

### 3.6. Therapy and prognosis

The clinical symptoms were relieved after anti-infection and symptomatic treatment, such as mannitol which was used to reduce intracranial pressure and diazepam or phenobarbital which was used to stop convulsions. Nine cases (6.9%) received intravenous steroid (dexamethasone) for 3–5 days in some patients who suffered from disturbance of consciousness. The follow-up period ranged from 2 to 69 months, with a median duration of 39 months. All the children showed normal neurodevelopment. There was full recovery of the lesions of MRI in all cases from 3 days to 50 days after the initial examinations. The median time of lesion disappearance (MRI) was 8 days (Table 1).

## 4. Discussion

RESLES is a new clinico-radiological syndrome that was originally reported as MERS by Tada et al. in 2004. In recent years, although there were more and more reports on RESLES, most of them were case reports, literature review, and small sample reports. Here, we retrospectively analyzed the clinical features of 130 children with RESLES in China, which is the largest case series available in the literature. In addition, children may have different clinical features due to different age of onset, and the 130 cases were divided into two groups: ≤ 3 years old group and >3 years old group. The clinical features of the two groups were compared in our study.

Of the 130 patients, the age of onset ranged from 8 to 153 months, with a median age of 32 months. Of these, 83 cases were younger than 3 years old and only 47 cases were older than 3 years old, indicating that RESLES is more likely to occur in young children. It is considered that the brain of children who are younger than 3 years old is immature and incomplete myelination. In addition to this, infants and young children are more susceptible to infection. Previous studies have shown that the most common cause of RESLES in children is infection (21). Consistent with previous studies, infection was the most common cause in our study. In our study, the vast majority of patients (97.7%) had prodromal symptoms of infection. More than half of the patients (54.6%) had preceding infections of the gastrointestinal tract, and 50 cases (38.5%) had preceding infections of the respiratory tract. In group A, more than half of the patients (72.3%) had preceding infections of the gastrointestinal tract. However, in group B, more than half of the patients (70.2%) had preceding infections of the respiratory tract. This indicates that patients at younger ages are more likely to have preceding infections of the gastrointestinal tract, and older patients are more likely to have preceding infections of the respiratory tract.

Various infectious agents have been reported to associate with RESLES in children, such as rotavirus, mycoplasma pneumoniae, influenza virus, salmonella, coxsackievirus, respiratory syncytial virus, streptococcus, and herpes zoster virus (7). In our study, 82 patients had clinically proven pathogen infection, including rotavirus in 44 cases (33.8%), mycoplasma pneumoniae in 14 cases (10.8%), influenza virus in 9 cases (6.9%), respiratory syncytial virus in 9 cases (6.9%), Epstein–Barr virus in 4 cases (3.1%), enterovirus in 1 case (0.8%), and SARS-CoV-2 in 1 case (0.8%), respectively. This indicates that rotavirus and mycoplasma pneumoniae are primary infectious agents, which are consistent with the results of the previous study (7). Our study also showed that infection of rotavirus was more common in younger patients and mycoplasma pneumoniae was more common in older patients, which has not been reported before. Although infection is the most common cause of the disease, the specific mechanism is unclear. Some scholars speculated that infection indirectly led to callosal lesions, which may be related to the immune inflammation mechanism after infection because pathogens were rarely found in the CSF (2, 7, 22).

In our study, the vast majority of patients (81.5%) developed seizures. Consistent with previous reports, seizure was the most common presenting symptom (23). In a subgroup of patients, seizures were accompanied by other neurological symptoms, including headache or dizziness (38 cases) and disturbance of consciousness (30 cases). In our study, in group A, almost all the patients (98.8%) developed seizures. However, in group B, only 24 cases (51.1%) developed seizures, which suggest that younger patients are more likely to develop convulsions. Our study also showed that older children were more likely to have symptoms with disturbance of consciousness and headache/dizziness. For those patients who are ≤ 3 years old, only part of patients are able to speak clearly when they have a headache/dizziness, which may be one reason why headache/dizziness symptoms are less likely to occur in younger age groups. However, the central nervous system manifestations of RESLES were non-specific, and they were mainly related to neurological syndrome. In our study, CwG is the most common neurological syndrome in the spectrum of RESLES. CwG was first proposed by the Japanese scholar Morooka in 1982 (24), and CwG is a syndrome that occurs in previously healthy infants and young children with mild gastroenteritis associated with afebrile seizure and is diagnosed after the exclusion of meningitis, encephalitis, encephalopathy, moderate-to-severe dehydration, electrolyte imbalance, or hypoglycemia, which usually has a benign prognosis (25, 26). At present, some scholars have reported that CWG can also cause RESLES, and RESLES may be related to the frequent seizures in patients with CwG (27). Other diseases can also cause RESLES, such as (23.1%) encephalitis/encephalopathy, (12.3%) febrile seizure (FS), (9.2%) epilepsy, and (10.8%) respiratory or digestive tract infection. Of the 12 patients with epilepsy, three patients did not have any prodromal symptoms of infections. The lesions of RESLES in these three patients with epilepsy may be related to convulsions, but the specific cause is still unknown. Our study also showed that CWG was more common in younger patients, while encephalitis/encephalopathy is more common in older patients.

In our study, MRI showed cytotoxic edema in SCC, which is sometimes associated with the entire corpus callosum and symmetrical cerebral white matter. These lesions were characterized by the hyperintense signal on T2WIs, FLAIR images, and DWIs and slightly hypointense on T1WIs, which is consistent with the results of the previous study (28). Our study also showed 125 cases (96.2%) were RESLES type-1 and only 5 cases (3.8%) were RESLES type-2, which suggests that RESLES type-1 is more common than RESLES type-2. Some scholars have speculated that type-1 and type-2 are two different stages of RESLES, and lesions of type-2 may have to pass through the phase of type-1 before it completely disappears (29). MRI of RESLES type-2 is similar to demyelinating diseases, such as ADEM. In general, the lesions on MRI with ADEM tend to be asymmetric and recovery is slow, which can take months. However, the lesions of RESLES type-2 tend to be symmetrical, and the recovery is faster. As reported in our study, MRI returned to normal in an average of approximately 8 days. Additionally, these lesions of RESLES should be differentiated from posterior reversible encephalopathy syndrome (PRES). PRES is usually characterized by hypertension, and the brain MRI shows abnormal signals in the bilateral posterior brain, such as mainly in the parietal occipital region. The lesions of PRES are associated with vasogenic brain edema. However, at present, it is generally agreed that the callosal lesions with reduced diffusion (low apparent diffusion coefficient value) in RESLES are caused by cytotoxic edema. Therefore, some scholars have termed these lesions cytotoxic lesions of the corpus callosum (CLOCCs) (30). The mechanism of cerebral edema in RESLES remains unclear. Possible mechanisms include intramyelinic edema due to the separation of myelin layers, interstitial edema in tightly packed fibers, and a transient inflammatory infiltrate (7, 30). In addition, hyponatremia may also be another mechanism of cerebral edema as our study showed that hyponatremia was presented in 30 cases (23.1%). In conclusion, the occurrence of RESLES is caused by multiple factors.

The treatment of RESLES is mainly to treat the neurological syndrome and carry out symptomatic treatment. In our study, the clinical symptoms were relieved after anti-infection and symptomatic treatment. Forty-three cases (33.1%) received mannitol which was used to reduce intracranial pressure. The increased intracranial pressure is based on clinical symptoms and signs, such as disturbance of consciousness, headache/dizziness, vomiting, frequent or prolonged convulsions, and papilledema, combined with measurement of CSF pressure. However, at present, there is no relevant literature to directly report the phenomenon of increased intracranial pressure in RESLES. The specific mechanism of increased intracranial pressure is still unknown. In addition, our retrospective study showed that the vast majority of patients (121/130 cases, 93.1%) who did not use hormones also recovered completely, and only 9 cases (6.9%) received intravenous dexamethasone for 3–5 days in some patients who suffered from disturbance of consciousness. All the children showed normal neurodevelopment. Therefore, further prospective randomized controlled studies are needed to determine whether hormones are beneficial in patients with RESLES. This is a limitation of our study. Of the 130 patients, 125 (96.2%) were RESLES type-1 and only 5 cases (3.8%) were RESLES type-2. Therefore, we were not able to study the correlation between RESLES type-1/2 and disease severity. This is another limitation of our study. Although the number of cases in our study was large, our study was a single-center retrospective study. Therefore, these cases were not representative of the epidemiology of RESLES in our region, and we were not able to calculate disease prevalence in the area, which also is a limitation of our study. Because of the limitations in our retrospective study, further prospective studies are needed to analyze the causes and treatment of various neurological syndrome in patients with RESLES, which is beneficial to the diagnosis and treatment of RESLES.

In conclusion, RESLES is a new clinico-radiological syndrome and has various etiologies. Infection was the most common cause. Infections of the gastrointestinal tract are common in ≤ 3 years old children, while infections of the respiratory tract are common in >3 years old children. Infections of rotavirus were more common in ≤ 3 years old children, and mycoplasma pneumoniae were more common in >3 years old children. Younger patients are more likely to develop convulsions, and older children were more likely to have symptoms with disturbance of consciousness and headache/dizziness. CWG was more common in ≤ 3 years old children, while encephalitis/encephalopathy is more common in >3 years old children. RESLES has characteristic MRI manifestations and a good prognosis.

## Data availability statement

The original contributions presented in the study are included in the article/supplementary material, further inquiries can be directed to the corresponding author.

## Ethics statement

The studies involving humans were approved by the Ethics Committee of Jiangxi Provincial Children's Hospital. The studies were conducted in accordance with the local legislation and institutional requirements. Written informed consent for participation in this study was provided by the participants' legal guardians/next of kin. Written informed consent was obtained from the individual(s), and minor(s)' legal guardian/next of kin, for the publication of any potentially identifiable images or data included in this article.

## Author contributions

This work was conceived by HC and ZT. Data was collected by XY, YC, and HW. The visualization work was performed and the manuscript was written by HC. XY, YC, HW, ZW, JZ, and ZT helped to revise manuscript and proposed constructive opinions. All authors contributed to the article and approved the submitted version.
